# SIX1 amplification modulates stemness and tumorigenesis in breast cancer

**DOI:** 10.1186/s12967-023-04679-2

**Published:** 2023-11-29

**Authors:** Liantao Guo, Faminzi Li, Hanqing Liu, Deguang Kong, Chuang Chen, Shengrong Sun

**Affiliations:** 1 Department of Breast and Thyroid Surgery, Renmin Hospital of Wuhan University, No. 238 Jiefang Road, Wuchang District, Wuhan, 430060 Hubei China; 2 Reproductive Medical Center, Renmin Hospital of Wuhan University and Hubei Clinic Research Center for Assisted Reproductive Technology and Embryonic Development, No. 238 Jiefang Road, Wuchang District, Wuhan, 430060 Hubei China

**Keywords:** Breast cancer, SIX1, Cancer stem cells, Tumorigenesis, Metastasis

## Abstract

**Background:**

Sine oculis homeobox homolog 1 (SIX1) is a transcription factor that has recently been identified as a crucial regulator of embryonic development and tumorigenesis. SIX1 is upregulated in different types of tumors, including breast cancer. However, the role and mechanism of SIX1 upregulation in breast cancer carcinogenesis remains uncertain.

**Methods:**

In this study, we utilized various databases such as UALCAN, TCGA, STRING, and Kaplan–Meier Plotter to investigate the mRNA expression, prognosis, transcriptional profile changes, signal pathway rewiring, and interaction with cancer stem cells of SIX1 in breast cancer. We also conducted both in vitro and in vivo experiments to validate its positive regulation effect on breast cancer stem cells.

**Results:**

Our findings demonstrated that the expression of SIX1 varies among different subtypes of breast cancer and that it upregulates breast cancer grading and lymph node metastasis. Besides, SIX1 participates in the rewiring of several cancer signaling pathways, including estrogen, WNT, MAPK, and other pathways, and interacts with cancer stem cells. SIX1 showed a significant positive correlation with breast cancer stem cell markers such as ALDH1A1, EPCAM, ITGB1, and SOX2. Moreover, our in vitro and in vivo experiments confirmed that SIX1 can promote the increase in the proportion of stem cells and tumor progression.

**Conclusions:**

Altogether, our results suggest that SIX1 plays an essential regulatory role in breast cancer's occurrence, and its amplification can be utilized as a diagnostic and prognostic predictor. The interaction between SIX1 and cancer stem cells may play a critical role in regulating breast cancer's initiation and metastasis.

**Supplementary Information:**

The online version contains supplementary material available at 10.1186/s12967-023-04679-2.

## Introduction

Breast cancer is a major type of cancer that affects a significant proportion of females, making up approximately 42% of all female cancer cases. It is a global health issue and causes substantial morbidity and mortality [1–4]. Given its heterogeneous nature and the limited treatment options currently available, such as radiotherapy, chemotherapy, and surgery, breast cancer remains a formidable challenge for both researchers and patients [4, 5]. Therefore, there is an urgent need to identify novel diagnostic biomarkers and therapeutic targets that play critical roles in the development and progression of breast cancer. Such discoveries hold the key to personalized precision therapy for individuals affected by this disease [5].

Homeobox genes are a class of transcription factors that play a crucial role in cellular development. These genes function as master regulators by controlling the expression of downstream target genes [6]. In humans, there are several families of homeoproteins, including the SIX gene family. The SIX family in humans can be categorized into three subclasses: SIX1/SIX2, SIX3/SIX6, and SIX4/SIX5 [7]. Among these, SIX1 is an important transcription factor that consists of a 281 amino acid long protein. Its N-terminus is composed of a 115 amino acid long SIX domain and a 60 amino acid long homeobox nucleic acid recognition domain [8–10]. The SIX domain acts as a mediator for protein interactions, while the homeobox nucleic acid recognition domain plays a role in DNA binding. Through its function as a transcriptional activator or repressor, SIX1 regulates numerous genes involved in development and differentiation [10–12]. Recent studies have shown that SIX1 is also involved in the etiology of various cancers, such as breast cancer, pancreatic cancer, prostate cancer, and ovarian cancer [10]. SIX1 contributes to the development of these cancers through mechanisms such as controlling cell cycle progression and promoting metastasis. However, the precise role and mechanism of SIX1 in regulating breast cancer carcinogenesis, as well as its interaction with other genes in promoting tumorigenesis, remain unclear. Moreover, a thorough investigation is needed to understand the relationship between SIX1 and clinical indexes, such as clinical stage, nodal metastasis status, ploidy, immune infiltration, gene alteration landscape, and the mRNA and protein landscape of SIX1.

Cancer stem cells (CSCs) represent a distinct population of cancer cells characterized by their ability to self-renew and differentiate into multiple cell types [13, 14]. They are also referred to as cancer stem cell-like cells, tumorigenic cells, tumor stem-like cells, and cancer- or tumor-initiating cells [13]. CSCs have been identified in various solid tumors, including breast cancer, colon cancer, skin squamous cell cancer, and glioblastoma [15, 13]. Moreover, extensive research has highlighted the significant role of CSCs in cancer initiation, recurrence, metastasis, and therapy resistance [13, 14]. Promising outcomes have been observed in numerous clinical experiments targeting CSCs [16], making them attractive therapeutic targets. Therefore, investigating the novel regulatory mechanisms of CSCs and their potential as therapeutic targets is of great interest. Previous studies have shown that SIX1, a transcription factor, enhances the expansion of phenotypic and functional CSCs in breast cancer [17], colorectal cancer [18] esophageal cancer [19] and phenotypic CSCs in pancreatic cancers [20]. However, the regulatory role of SIX1 on CSCs in breast cancer primarily pertains to the luminal subtype, while its mechanisms in other subtypes remain largely unexplored. Consequently, it is crucial to determine whether SIX1 can also regulate CSCs in other subtypes of breast cancer and elucidate the underlying mechanisms involved. Such insights will pave the way for the development of novel therapeutic approaches against breast cancer.

In this study, we employed diverse databases to investigate changes in gene expression, prognostic significance, ploidy variations, immune infiltration, gene alteration landscape, interacting genes, and altered signaling pathways associated with SIX1 during breast cancer tumorigenesis. Through a combination of in vitro and in vivo experiments, we validated the regulatory role of SIX1 in promoting the growth of breast cancer stem cells (BCSCs). Our findings aim to contribute to the identification of novel biomarkers and therapeutic targets for breast cancer.

## Materials and methods

### Differential mRNA expression of SIX1 and its relationship with prognosis

We obtained the standardized pan-cancer dataset, TCGA TARGET GTEx (PANCAN, N = 19,131, G = 60,499), from the UCSC (https://xenabrowser.net/) database. Specifically, we extracted the expression data of the ENSG00000126778 (SIX1) gene from various samples, including solid tissue normal, primary solid tumor, primary tumor, normal tissue, primary blood derived cancer—bone marrow, primary blood derived cancer—peripheral blood samples. To ensure consistency, we performed a log2(x + 0.001) transformation for each expression value. We further filtered out cancer species with less than 3 samples, resulting in a final set of 34 cancer species with their corresponding expression data. To investigate the expression of SIX1 in the four major subtypes of breast cancer (luminal A, luminal B, HER2-positive, and triple-negative breast cancer), we downloaded RNA-sequencing expression (level 3) profiles and corresponding clinical information for breast cancer from the TCGA dataset (https://portal.gdc.com). Additionally, we obtained the current-release (V8) GTEx datasets from the GTEx data portal website (https://www.gtexportal.org/home/datasets). To explore the association between SIX1 mRNA expression and prognosis, we utilized two databases: Kaplan–Meier plotter (http://kmplot.com/analysis/) [21] and UALCAN (http://ualcan.path.uab.edu) [22]. Firstly, we used Kaplan–Meier plotter to analyze the overall survival (OS) curves of patients with high and low SIX1 expression in the aforementioned four major subtypes of breast cancer. Patients were classified as having high or low SIX1 expression based on the median SIX1 expression. Subsequently, we employed the UALCAN database to investigate the differential mRNA expression of SIX1 in various tumor histologies of breast cancer, as well as individual cancer stages and nodal metastasis statuses.

### The relationship between the expression of SIX1 and ploidy and immune infiltration

The TCGA Pan-Cancer (PANCAN) dataset, consisting of 10,535 cases and 60,499 genes, was downloaded from the UCSC database (https://xenabrowser.net/). This dataset provides a standardized and comprehensive collection of pan-cancer data. To investigate the relationship between SIX1 expression, ploidy, and immune infiltration, we specifically extracted the expression data of the ENSG00000126778 (SIX1) gene from multiple samples. To ensure the reliability of the data, we focused on primary blood-derived cancers, including peripheral blood and primary tumor samples. Ploidy data for each tumor were obtained from a previous study [23]. We then integrated the ploidy data with the gene expression data. To facilitate analysis, a log2(x + 0.001) transformation was applied to each value. To further streamline the analysis, we excluded cancer species with fewer than 3 samples within each specific cancer type. As a result, we obtained expression data for 37 different cancer species. Subsequently, we explored the relationship between SIX1 expression and two key indicators: stromal score (which reflects the presence of stroma in tumor tissue) and immune score (which denotes the infiltration of immune cells in tumor tissue) [24]. For this analysis, we utilized the SangerBox website (http://vip.sangerbox.com/home.html), a valuable online platform specifically designed for TCGA data analysis. Additionally, using the SangerBox website, we further investigated the relationship between SIX1 expression and various immune cell types, including B cells, M1 macrophages, M2 macrophages, monocytes, neutrophils, natural killer cells, CB4 + T cells, CB8 + T cells, Tregs, and dendritic cells.

### The gene alteration landscape of SIX1

We utilized cBioportal (https://www.cbioportal.org) [25] and TCGA (https://portal.gdc.cancer.gov/) databases to investigate gene alterations related to SIX1 in patients with breast cancer. To obtain the gene alterations from cBioportal, we first selected the Breast Invasive Carcinoma (TCGA, PanCancer Atlas) and selected genomic profiles, including mutations, putative copy-number alterations from GISTIC, and mRNA expression z-scores relative to all samples (log RNA Seq V2 RSEM). We selected all available samples as our patient/case set, then queried SIX1 to obtain information on genetic alterations, including their proportion and type, within breast cancer patients and subtypes. We then downloaded RNA-sequencing expression (level 3) profiles, genetic mutation data, and corresponding clinical information for breast cancer from the TCGA. The mutation data were downloaded and visualized using the maftools package in R software. Genes with higher mutational frequency detected in breast cancer patients were displayed as a histogram.

### The mRNA and protein landscape of SIX1

RNA-sequencing expression profiles and associated clinical information for breast cancer were downloaded from the TCGA database. Differential mRNA expression was studied using the limma package in R software. Patients were grouped based on the expression levels of SIX1, with the experimental group comprising patients in the top 25% and the control group comprising patients in the bottom 25%. A threshold of "Adjusted P < 0.05 and Fold Change > 1.5 or Fold Change < − 1.5" was defined to identify differentially expressed mRNAs, and genes with differential expression were selected for further analysis. To further analyze the differentially expressed genes, the STRING database (https://string-db.org/) [26] was utilized to construct a protein–protein interaction network associated with SIX1. To investigate potential targets' function, gene functional enrichment analysis was conducted using Gene Ontology (GO) and Kyoto Encyclopedia of Genes and Genomes (KEGG) Enrichment Analysis. The ClusterProfiler package (version: 3.18.0) in R software was used to analyze GO functions and KEGG pathway enrichments to improve the understanding of mRNA carcinogenesis. Box plots were created using the ggplot2 package in R, while heatmaps were generated using the pheatmap package.

### The relationship of SIX1 with cancer stem cells

We downloaded the TCGA Pan-Cancer (PANCAN, N = 10,535, G = 60,499) dataset, which is a standardized pan-cancer dataset, from the UCSC (https://xenabrowser.net/) database. Specifically, we extracted the expression data of the ENSG00000126778 (SIX1) gene from various samples. To ensure data quality, we selected only primary blood derived cancer—peripheral blood and primary tumor samples. Subsequently, we utilized the OCLR algorithm, as proposed by Malta et al. [27], to calculate the RNAss stemness score based on mRNA features. We then integrated the tumor stemness scores and gene expression data of the samples. Finally, to ensure statistical power, we removed cancer types with fewer than three samples, ultimately obtaining expression data for 37 different cancers. In each type of tumor, we computed their Pearson correlation coefficients. In the present study, we investigated the association between SIX1 and stem cell markers in breast cancer patients using data obtained from the TCGA database. Pearson correlation analysis was performed to explore the relationship between these factors.

### Cell culture

MCF7 derivative cell lines were described previously [28]. The 66cl4 cell line, generously provided by Prof. Kongming Wu from Tongji Medical College, Huazhong University of Science and Technology, was employed in this study. We generated MCF7-SIX1 cell line by overexpressing SIX1 in MCF7 cells for convenience in description. MCF7-NC was used as the negative control for comparison. To knock down Six1 expression in 66cl4 cells, we targeted two sites of Six1 and generated two knockdown cell lines, named 66cl4-shSix1 KD1 and 66cl4-shSix1 KD2, respectively. The negative control for knockdown experiments was 66cl4-SCR. All cells were cultured in Dulbecco’s modified Eagle’s medium (DMEM) supplemented with 10% fetal bovine serum and 1% penicillin/streptomycin at 37 °C in 5% CO_2_.

### Western blot analysis

We electrophoresed equal amounts of lysates, ranging from 30 to 50 μg, onto polyvinylidene difluoride membranes. Subsequently, the membranes were blocked using TBST with 5% milk and probed with primary antibodies, specifically β-actin (1:5000; Abmart, M20011F), SIX1 (1:1000, Cell Signaling, D5S2S), SOX2 (1:1000, abcam, ab92494), Oct4 (1:1000, abcam, ab181557), c-Myc (1:1000, Abmart, TA0358S), EPCAM (1:1000, huabio, EM1111), ALDH1A1 (1:1000, abcam, ab52492), ITGB1 (1:1000, abcam, ab179471), p-STAT3 (1:1000, Abmart, T56566F), and STAT3 (1:1000, Abmart, T55292F), overnight at 4 °C. After washing thrice with TBST, the membranes were incubated for 1.5 h at room temperature with secondary antibodies, including goat anti-rabbit IgG-HRP (1:10,000, Proteintech, SA00001-2) and goat anti-mouse IgG-HRP (1:10,000, Proteintech, SA00001-1).

### Quantitative real-time PCR (RT-qPCR)

RNA was prepared using the RNAeasy™ Animal RNA Isolation Kit with Spin Column (Beyotime, R0026). cDNA was reverse transcribed from 1 μg total RNA using the HiScript® III All-in-one RT SuperMix Perfect for qPCR (Vazyme, R333-01). RT-qPCR was performed with the Taq pro Universal SYBR qPCR Master Mix (Vazyme, Q712-02). The primer sequences for RT-qPCR were as follows: Six1 (mouse): (forward) 5′-CAAGAACGAGAGCGTGCTCAAGG-3′, (reverse) 5′-GGTGATTGTGAGGCGAGAACTGG-3′; SIX1 (human): (forward) 5′-CAAGAACGAGAGCGTACTCAAGGC-3′, (reverse) 5′-GGTGGTTGTGAGGCGAGAACTG-3′; Oct4 (mouse): (forward) 5′-CATTGAGAACCGTGTGAGGTGGAG-3′, (reverse) 5′-GCGATGTGAGTGATCTGCTGTAGG-3′;OCT4 (human): (forward) 5′-GTGGTCCGAGTGTGGTTCTGTAAC-3′, (reverse) 5′-CCCAGCAGCCTCAAAATCCTCTC-3′; Sox2 (mouse): (forward) 5′-CAGCATGTCCTACTCGCAGCAG-3′, (reverse) 5′-CTGGAGTGGGAGGAAGAGGTAACC-3′; SOX2 (human): (forward) 5′-CAGCATGTCCTACTCGCAGCAG-3′, (reverse) 5′-CTGGAGTGGGAGGAAGAGGTAACC-3′; Aldh1a1 (mouse): (forward) 5′-ATGGTTTAGCAGCAGGACTCTTCAC-3′, (reverse) 5′-CCAGACATCTTGAATCCACCGAAGG-3′; ALDH1A1 (human): (forward) 5′-ACGCCAGACTTACCTGTCCTACTC-3′, (reverse) 5′-TCTTGCCACTCACTGAATCATGCC-3′; CD44 (mouse): (forward) 5′-CTCAAGTGCGAACCAGGACAGTG-3′, (reverse) 5′-ATCAGAGCCAGTGCCAGGAGAG-3′; CD44 (human): (forward) 5′-TCTACAAGCACAATCCAGGCAACTC-3′, (reverse) 5′-ATGGGAGTCTTCTTTGGGTGTTTGG′; The β-actin primers (Sangon Biotech, B661302) and β-ACTIN primers (Sangon Biotech, B661102) were purchased from Sangon.

### Mammosphere formation and self‑renewal capability assay

Cells were dissociated into single cells by 0.05% trypsin‐EDTA solution and plated into Corning ultralow attachment culture dish (Corning, 3471) at a density of 2 × 10^3^ viable cells per milliliter in primary culture. Cells were grown in a serum‐free DMEM medium supplemented with B27 (Gibco, 17,504,044), 20 ng/mL Epidermal Growth Factor (PeproTech, 315-09/AF-100-15), 20 ng/mL Basic fibroblast growth factor (PeproTech, AF-450–33/100-18B), 2 μg/mL heparin (MCE, HY-17567). For MCF‐7 cells, mammospheres were kept in culture 7 days. Whereas 66cl4 mammospheres were kept in culture 5 days. To assess self-renewal capacity, mammospheres (diameter > 50 µm) were manually enumerated and representative images captured using an OLYMPUS IX71 microscope (Tokyo, Japan). Mammosphere-forming efficiency was calculated as follows: (number of mammospheres per well/number of cells seeded per well) × 100.

#### Flow cytometry analysis

To detect the stem cell subpopulations, the following antibodies were used: APC anti-mouse-CD24 (Biolegend, 101,813, 1:167 dilution), PE anti-mouse-CD49f (Biolegend, 313,612, 1:167 dilution), APC anti-human-CD24 (Biolegend, 311,117, 1:167 dilution), PE anti-human-CD44 (Biolegend, 103,007, 1:167 dilution). A total of 1 × 10^6^ cells were incubated with antibodies in the dark at 4 °C for 30 min. Cells were washed and re-suspended in 500 µl of PBS and analyzed using a flow cytometer (Beckman Coulter, CytoFLEX).

#### Aldehyde dehydrogenase (ALDH) activity

Cells were first placed on ice then ALDH was detected by an ALDH test kit (Solarbio, BC0755) as indicated by the manufacturer. All ALDH activities were evaluated using a microplate reader (PerkinElmer, Ensight) at 340 nm by measuring the production of NAD + . Higher optical density (OD) values indicate stronger activity.

#### Cell proliferation assay

The influence of SIX1 on cancer cells viability were determined with Cell Counting Kit 8 (CCK-8) assay (Biosharp, BS350B) according to the manufacturer’s instructions. For CCK-8 assay, 1000 cells of various 66cl4 and 2000 cells of various MCF7 were plated in 96-well plates. Day 0 time point was measured 6 h post plating. Following 24-, 48-, 72- or 96-h of incubation, day 1–4 time points were analyzed. 10 μl of CCK-8 was added to each well and incubated at room temperature for 60 min and luminescence was measured by using a microplate reader (PerkinElmer, Ensight) at 450 nm.

#### Tumor-bearing model and imaging

Prior to the commencement of experiments, female BALB/c mice (6–8 weeks old) were provided with ad libitum access to food and water. All animal studies were meticulously reviewed and granted ethical clearance by the Laboratory Animal Welfare & Ethics Committee of Renmin Hospital at Wuhan University (Issue No. 20200702). It should be noted that all animal experiments strictly adhered to the guidelines articulated in the Guide for the Care and Use of Laboratory Animals developed by the Institute of Laboratory Animal Research. Firstly, a total of 18 mice were randomly assigned to three distinct groups. Specifically, cells harvested from 66cl4-SCR-luc, 66cl4-shSix1 KD1-luc, and 66cl4-shSix1 KD2-luc were resuspended in serum-free medium at a density of 1 × 10^6^ cells per 100 μl. Subsequently, tumor cells were administered orthotopically into the fourth mammary fat pad of the mice. The tumor volume and luminescence signals were monitored on a weekly basis via an in vivo imaging system (PerkinElmer, IVIS Spectrum). To assess the impact of Six1 knockdown on the tumorigenic capacity of 66cl4 cells in vivo, we performed gradient dilution experiments by injecting varying numbers of cells for comparison. A total of 54 mice were randomly divided into three groups. Cells obtained from 66cl4-SCR-luc, 66cl4-shSix1 KD1-luc, and 66cl4-shSix1 KD2-luc were suspended in serum-free medium at a density range of 1*10^4^ to 1*10^6^ cells per 100 μl. Subsequently, the tumor cells were orthotopically administered into the fourth mammary fat pad of the mice. Tumor sizes and weights were monitored starting from 10 days post-injection.

#### In vivo expression of cancer stem cell markers in tumors

Tumors from each aforementioned group were fixed with 4% paraformaldehyde at room temperature for 24 h, followed by paraffin embedding and sectioning into 5 µm thick slices. Deparaffinization of paraffin sections was carried out using standard techniques, and sodium citrate antigen retrieval was performed. The sections were permeabilized for 15 min with 0.1–0.25% Triton X-100 and blocked for 30 min with 10% goat serum. Subsequently, different sections were incubated overnight at 4 °C with specific primary antibodies for Oct4 (1:1000, abcam, ab181557), Sox2 (1:100, abcam, ab92494), Aldh1a1 (1:100, abcam, ab52492), Epcam (1:20,000, abcam, ab213500), and Itgb1 (1:1000, abcam, ab179471). Following this, goat anti-rabbit Immunoglobulin G H&L (HRP) (1:100, abcam, ab205718) was incubated at room temperature for 1 h. Finally, all slides were stained with 300 nM DAPI for 5 min at room temperature. Fluorescence microscopy at × 40 magnification was used to observe images in each sample.

#### Statistical analysis

Statistical analyses were performed using GraphPad Prism 9.0 software (GraphPad Software). The Student's t-test was employed for two-group comparisons, while one-way ANOVA non-parametric was used for comparisons involving three or more groups. Proliferation data and tumor growth curves were subjected to two-way ANOVA analysis. Statistical significance was indicated by P values < 0.05. Detailed P-values can be found in the figures.

## Results

### Interrelation of the changes of the expression of SIX1 mRNA with the clinicopathological parameters and the clinical prognosis of the breast cancer patients.

**Fig. 1:**
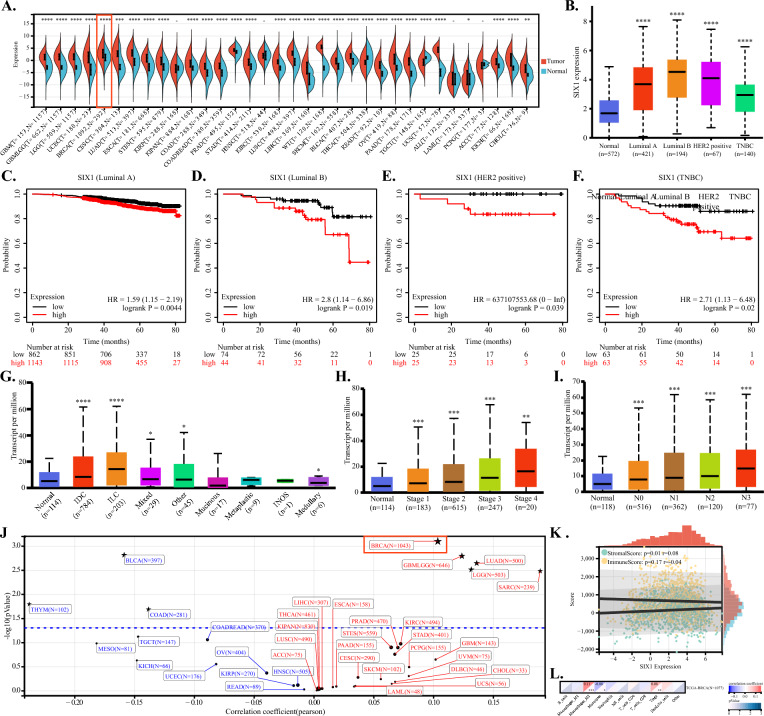
The expression of SIX1 and its association with the clinical prognosis of breast cancer patients. **A** Differential expression of SIX1 across different cancer types based on pan-cancer analysis. **B** Box plots illustrating expression analysis of SIX1 in four major subclasses of breast cancer patients. **C**–**F** Kaplan–Meier survival curves of overall survival (OS) based on SIX1 expression in the four major subclasses of breast cancer patients (log-rank test, P < 0.01). **G**–**I** Box plots depicting SIX1 expression analysis according to breast cancer histology, stages, and nodal metastasis, respectively. **J** Scatter plot demonstrating the correlation between SIX1 expression and ploidy in each tumor, with positive correlation shown in red and negative correlation shown in blue. **K** Correlation scatter plot indicating the relationship between SIX1 expression and stromal score and immune score in breast cancer. A value > 0 represents a positive correlation, while an r < 0 represents a negative correlation. **L** Heat map displaying the correlation between SIX1 expression and immune cell infiltration in breast cancer patients. The square above each cell type is divided into two triangles. The color of the top left triangle represents the correlation, where red indicates positive correlation and blue indicates negative correlation. The color of the bottom right triangle represents statistical significance, with lighter colors indicating greater statistical significance. *P < 0.05, **P < 0.01, ***P < 0.001

To gain a comprehensive understanding of SIX1 expression across various tissues and its correlation with clinicopathological features in breast cancer patients, we utilized an online database. We extracted the standardized pan-cancer dataset, TCGA TARGET GTEx (PANCAN, N = 19,131, G = 60,499), from UCSC (https://xenabrowser.net/). Differential expression between normal and tumor samples was calculated for each tumor type, and differences in significance were analyzed using unpaired Wilcoxon Rank Sum and Signed Rank Tests. As illustrated in Fig. 1A, significant up-regulation of SIX1 was observed in 28 tumors, including breast cancer (Tumor: 2.62 ± 2.27, Normal:1.05 ± 1.77, P = 2.8e−29), while down-regulation was noted in 2 tumors. Notably, Fig. 1B demonstrates that SIX1 is highly expressed in all breast cancer subtypes compared to normal tissues, with the highest expression levels seen in the luminal B subtype. To further investigate the correlation between SIX1 expression and overall survival (OS) in four major subtypes of breast cancer patients, we utilized the KMPlot (http://kmplot.cm/analysis) database. As depicted in Fig. 1C–F, a significant association between SIX1 expression and OS in breast cancer patients was observed. Patients with high SIX1 expression in all four subtypes of breast cancer had a worse prognosis compared to those with low SIX1 expression. We also examined the difference in SIX1 expression among different histology subtypes of breast cancer. Figure 1G shows significantly higher expression of SIX1 in invasive ductal carcinoma (IDC) and invasive lobular carcinoma (ILC) compared to normal tissues, while mixed, medullary, and other undefined histological subtypes exhibited slightly higher expression levels. Figure 1H demonstrates a trend towards increased median expression levels of SIX1 as breast cancer progresses to advanced stages, while Fig. 1I suggests a positive relationship between the number of lymph node metastases in breast cancer patients and elevated median expression levels of SIX1. The collective evidence presented in Fig. 1H, I indicates that SIX1 may have an accelerating effect on the progression and metastasis of breast cancer. Additionally, we calculated the Pearson correlation between SIX1 expression and ploidy in each tumor and observed a significant positive correlation in 5 tumors, including BRCA (N = 1043(R = 0.103690910291636, P = 0.000797159290285103), and a significant negative association in 4 tumors. This suggests that SIX1 is closely related to polyploidy and chromosomal instability in breast cancer (Fig. 1J). Furthermore, in breast cancer, SIX1 expression is significantly and positively correlated with Stromal score, indicating an increase in other tumor microenvironment components such as fibroblasts. Conversely, there is a tendency for Immune Score to decrease, suggesting that SIX1 expression may reduce the infiltration of immune cells (Fig. 1K). Nevertheless, despite the observed reduction in immune cell infiltration, our findings revealed an intriguing phenomenon whereby M2 macrophages and Treg cells exhibited increased infiltration concomitant with elevated SIX1 expression (Fig. 1L).

### The gene alteration landscape of SIX1

**Fig. 2:**
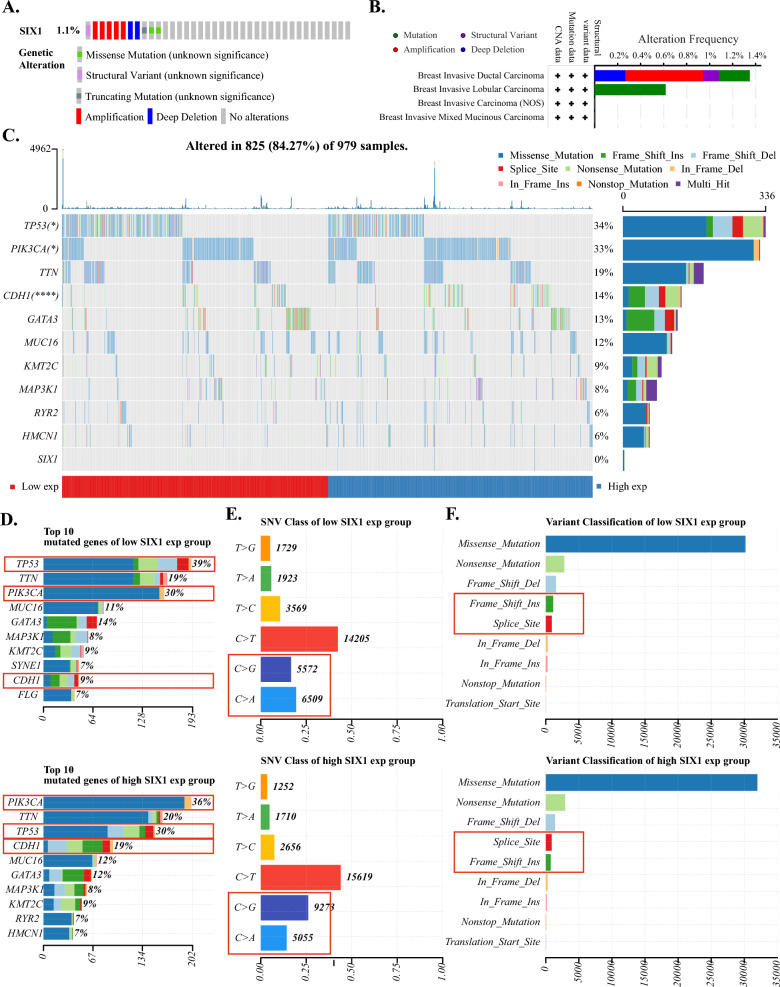
The genetic alteration landscape of SIX1. **A**, **B** Schematic diagrams depicting the proportion and type of genetic alterations of SIX1 in breast cancer patients and breast cancer subclasses, respectively, in cBioportal database. **C** Oncoplot shows the somatic landscape of breast tumor cohort. Genes are ordered by their mutation frequencies, samples are ordered by expression of SIX1, as indicated by the annotation bar (bottom). Side bar plot shows the -log10-transformed q-values, as estimated using MutSigCV. Waterfall plot shows mutation information for each gene for each sample. Color annotation of various cancer types are shown at the bottom. The barplot above the legend shows the number of mutation burden. **D** and **E**. Cohort summary plot shows the distribution of variants according to variant classification, and SNV class. **F** Stacked bar graph shows the top ten mutated genes

To investigate the gene structure and transcriptional alterations of SIX1, cBioportal (https://www.cbioportal.org) database was utilized. Regarding genetic changes in SIX1, Fig. 2A, B showed that SIX1 gene was modified in 1.1% of the patients with amplification being the primary type of alteration in breast cancer. Among various subtypes of breast cancer, Breast Invasive Ductal Carcinoma exhibited the highest mutation frequency of SIX1 followed by Breast Invasive Lobular Carcinoma; whereas, Breast Invasive Mixed Mucinous Carcinoma and Breast Invasive Carcinoma (NOS) demonstrated minimal mutational changes of SIX1. Though, amplification was the dominant type of mutation in all breast cancer subtypes, accounting for nearly half the frequency. Figure 2C presented the somatic landscape of breast tumor cohort. Different expression of SIX1 appeared to be associated with distinct gene alterations. According to Fig. 2C, D PIK3CA, TP53, CDH1 had substantially different mutated status between low and high SIX1 expression groups. The frequency of mutated PIK3CA and CDH1 were higher in the high expression group, while TP53 was not. Mutated PIK3CA, TP53, CDH1 may play a certain role in breast cancer progression influenced by SIX1. As per Fig. 2E, among the low SIX1 expression group, the mutation of C > A ranked second, larger than the mutation of C > G ranked third. In contrast, the mutation of C > G was the second most common alteration in the high SIX1 expression group, larger than the mutation of C > A. There were some differences in the Variant Classification presented in Fig. 2F between these two groups as well. The mutation of Frame_Shift_Ins ranked fourth, larger than the fifth-ranked Splice_Site mutation in the low SIX1 expression group, while Splice_Site ranked fourth in the high SIX1 expression group, larger than the fifth-ranked Frame_Shift_Ins mutation. These differences might result from changes in SIX1 expression, and further investigations are required to determine their impact on breast cancer progression, which will be beneficial for us to gain a better understanding of the functions of SIX1.

### The mRNA and protein landscape of SIX1

**Fig. 3:**
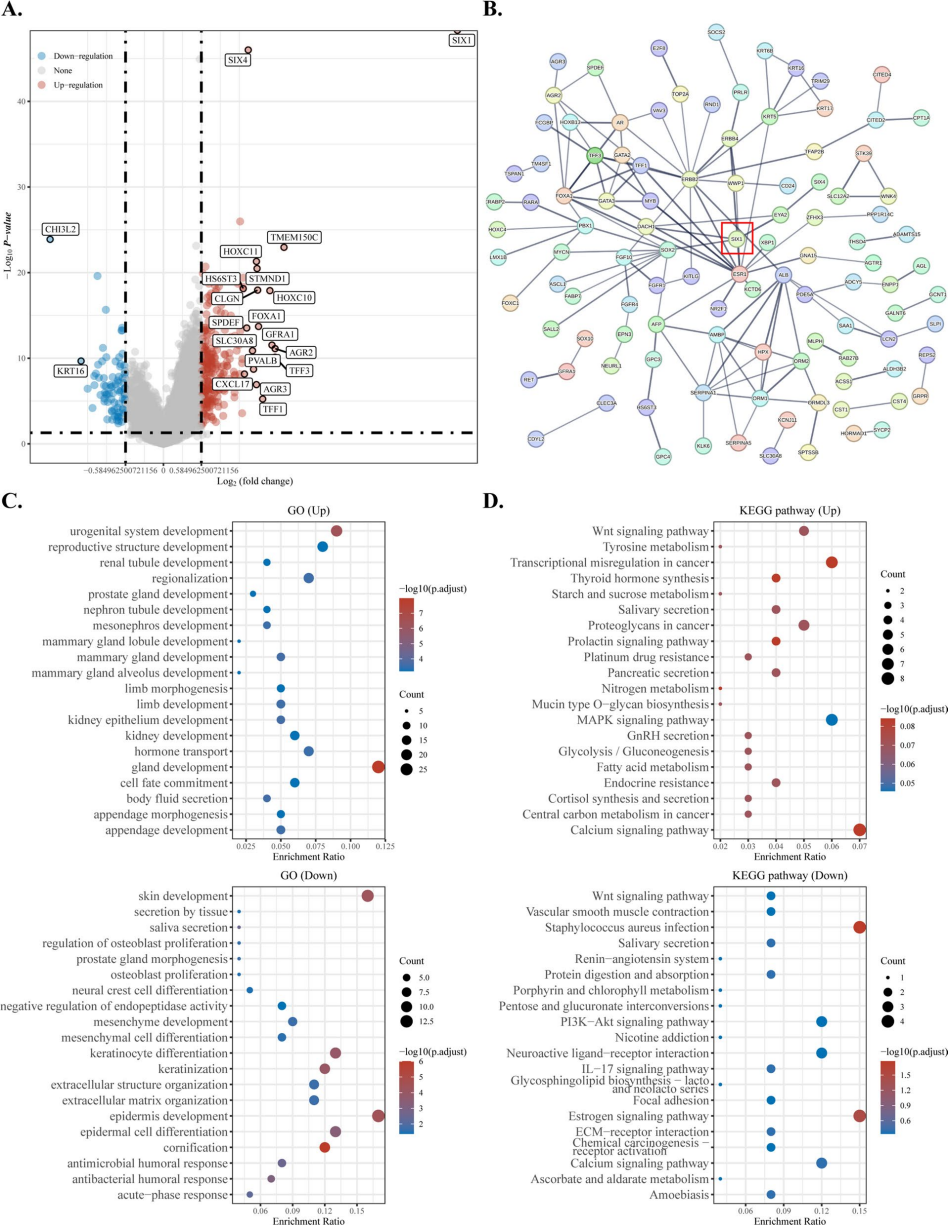
The mRNA and protein landscape of SIX1. **A** The volcano plot was constructed using the fold change values and P-adjust. Red dots indicate upregulated genes; blue dots indicate downregulated genes; grey dots indicate not significant. **B** Protein–protein interaction network revealed the interactions among proteins translated from differentially expressed genes, identified through screening based on SIX1 expression levels. **C** and **D** GO analysis of potential targets of mRNAs, the biological process, cellular component, and molecular function of potential targets were clustered based on ClusterProfiler package in R software (version: 3.18.0). The enriched KEGG signaling pathways were selected to demonstrate the primary biological actions of major potential mRNA. The abscissa indicates gene ratio and the enriched pathways were presented in the ordinate. Colors represent the significance of differential enrichment, the size of the circles represents the number of genes analyzed, the larger the circle, the greater the number of genes. In the enrichment result, P < 0.05 or FDR < 0.05 is considered to be a meaningful pathway (enrichment score with − log10 (P) of more than 1.3)

Tumorigenesis can be interpreted as the outcome of an imbalanced expression of signal pathways. SIX1, an important downstream regulator of the signal pathway, is critical to understand the occurrence of breast cancer. Therefore, comprehending the rewiring pathway of SIX1 is indispensable. Breast cancer data from TCGA was downloaded and screened for differentially expressed genes between high and low SIX1 expression groups. Upregulated genes including SIX4, SLC4A8, TMEM150C, HOXC11, ZFHX3, STMND1, KIAA1958, and RIIAD1 were identified in the high-expression group compared to the low-expression group, as well as downregulated genes including CHI3L2, GASK1A, TTC22, FXYD5, and GNA15 (Fig. 3A). Details of differential gene analysis are presented in Additional file 1: Table S1. To further explore the relationship between these genes and SIX1, they were input into the STRING database to construct a protein–protein interaction network. Figure 3B shows that SIX1 is in a relatively central area and interacts with most of the proteins.

The concomitant changes in expression levels of these genes provide insights into the putative functions of SIX1. Therefore, GO and KEGG enrichment analyses were conducted on these differentially expressed genes to annotate their functions and investigate the role of SIX1. Figure 3C shows that these genes contributed to gland development, particularly mammary gland development, including mammary gland lobule development and mammary gland alveolus development, skin cell differentiation, mesenchymal cell differentiation, and mesenchyme development. This result suggests that SIX1 may contribute to the progression of breast cancer through regulation of mammary gland development and epithelial-mesenchymal transition (EMT). Figure 3D indicates that not all KEGG enrichment entries were statistically significant, but they still have the potential to contribute to a deeper understanding of the functionality of SIX1. These genes may be enriched in classical signaling pathways, such as the Wnt signaling pathway, MAPK signaling pathway, calcium signaling pathway, PI3K-Akt signaling pathway, and IL-17 signaling pathway. Additionally, these genes may regulate tyrosine metabolism, starch and sucrose metabolism, nitrogen metabolism, glycolysis/gluconeogenesis, fatty acid metabolism, and central carbon metabolism in cancer, which have been reported to be associated with cancer. Furthermore, we found genes that may contribute to therapy resistance, including platinum drug resistance.

### Correlation between SIX1 and breast cancer stem cells

**Fig. 4:**
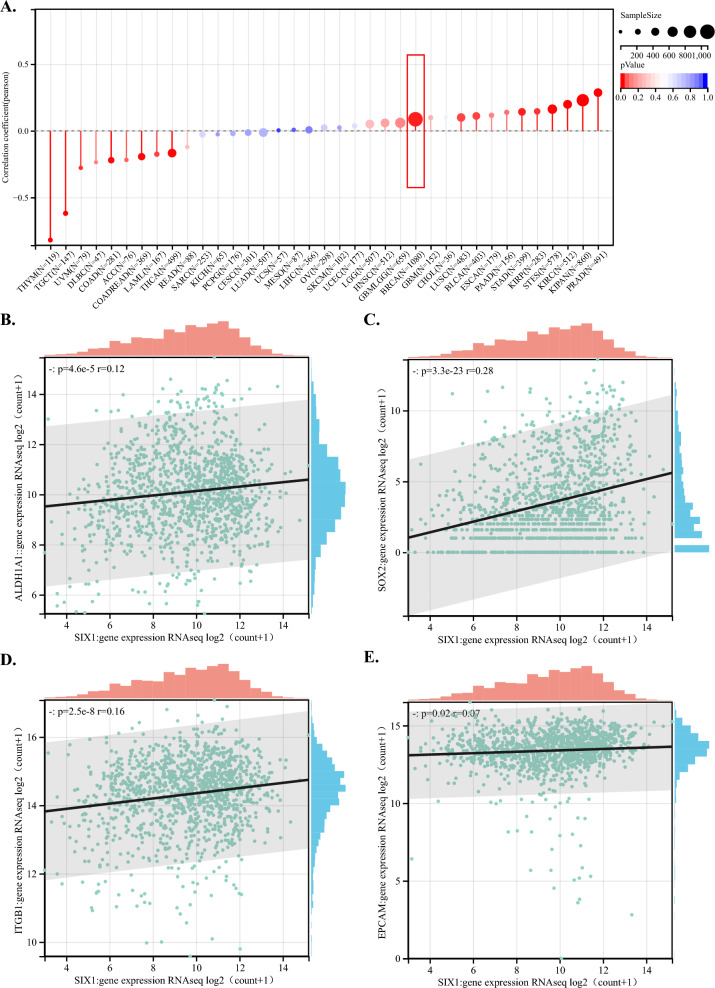
Relationship between SIX1 and cancer stem cells. **A** The lollipop plot depicts the correlation between SIX1 and tumor stemness in pan-cancer. The x-axis represents different types of tumors, while the y-axis illustrates the correlation between SIX1 expression and RNA stemness scores. Positive values indicate a positive correlation, whereas negative values indicate a negative correlation. Colors represent the significance of differential cancer, the size of the circles represents the number of samples analyzed, the larger the circle, the greater the number of samples. Correlation scatter plots illustrating the relationship between SIX1 expression and ALDH1A1, SOX2, EPCAM, and ITGB1 in breast cancer (panels **B** to **E**). A value of 0 denotes a positive correlation, while a value of r < 0 indicates a negative correlation. P < 0.05 is considered to be a meaningful correlation

Based on the enrichment results mentioned above, we speculate that SIX1 is involved in regulating various biological processes such as breast development, EMT regulation, metabolism regulation and drug resistance. These processes have been reported to be connected to stem cells in the literature. Thus, it raises an important question: does SIX1 have a regulatory relationship with stem cells? Can SIX1 modulate breast cancer progression by regulating stem cells? Further studies are urgently needed to address these questions. In Fig. 4A, we retrieved the standardized pan-cancer dataset, TCGA Pan-Cancer (PANCAN, N = 10,535, G = 60,499), from UCSC (https://xenabrowser.net/). Specifically, we extracted the expression data of ENSG00000126778 (SIX1) in each sample and obtained the RNA stemness scores calculated through mRNA features for each tumor. We calculated the Pearson correlation coefficients in each tumor type and observed significant correlations in 16 tumors. Notably, we found positive correlations in nine tumors, including breast cancer (R = 0.0864774535192319, P = 0.00445542350727206), and negative correlations in seven tumors. Furthermore, in breast cancer patients, we explored the correlation between SIX1 and common tumor.

stem cell markers and found significant positive correlations with ALDH1A1, SOX2, ITGB1, EPCAM, among others (Fig. 4B–E). Therefore, our preliminary findings suggest that SIX1 may regulate stem cells in breast cancer positively, but further experimental validation is needed.

### SIX1 expression in breast cancer cells can influence the expression of stem cell markers

**Fig. 5:**
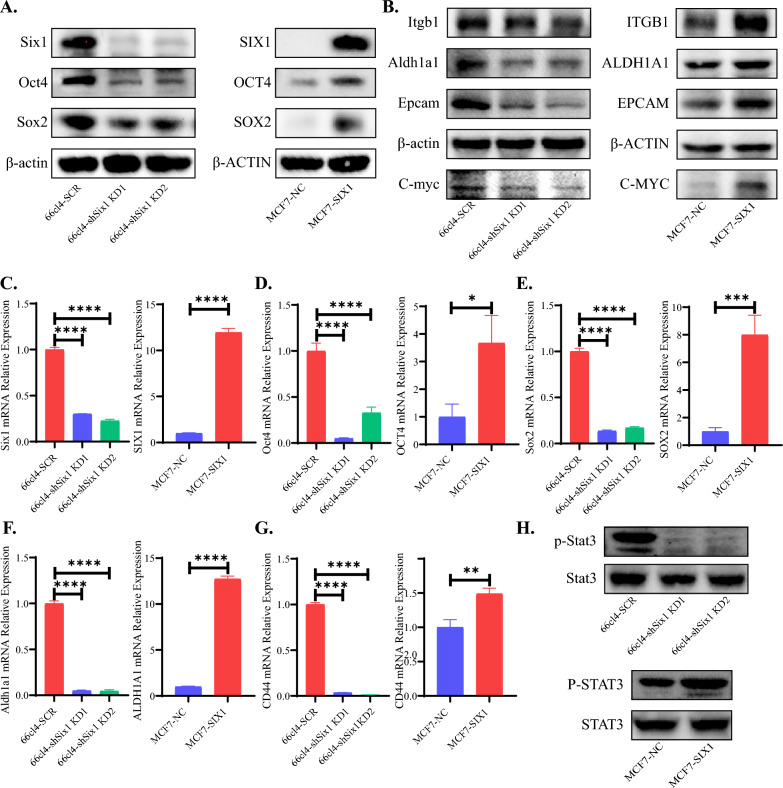
In vitro alterations in breast cancer stem cell associated markers and corresponding changes in the STAT3 signaling pathway in response to modulation of SIX1 expression levels. **A**, **B** and **H** Representative western blot analysis of whole cell lysates from 66cl4-SCR, 66cl4-shSIX1 KD1, 66cl4-shSIX1 KD2, MCF-7-NC and MCF-7-SIX1 cells. **C**–**G** qRT-PCR analyses of SIX1 and breast cancer stem cell associated markers using 1 μg RNA from 66cl4-SCR, 66cl4-shSIX1 KD1, 66cl4-shSIX1 KD2, MCF-7-NC and MCF-7-SIX1 cells. Gene expression is normalized to β-actin mRNA (n = 3)

To further validate the impact of SIX1 on breast cancer stem cells and ensure the integrity and reliability of our experiments, we utilized two cell lines: murine breast cancer cell line 66cl4 and human breast cancer cell line MCF-7. As the expression level of Six1 and the degree of malignancy were relatively high in 66cl4 cells, we knocked down Six1 in this cell line to investigate its effect on BCSC proportion. Conversely, as the expression level of SIX1 and the degree of malignancy showed moderate levels in MCF-7, we overexpressed SIX1 in this cell line to explore its impact on BCSC proportion. The successful establishment of our cell models was confirmed by Fig. 5A, C depicting Six1 knockdown in 66cl4 cells and SIX1 overexpression in MCF7 cells, respectively. Notably, we observed a concurrent decrease in SOX2, OCT4, and ALDH1A1 at both the protein and mRNA levels upon Six1 knockdown in 66cl4 cells, whereas their expression increased upon SIX1 overexpression in MCF7 cells (Fig. 5A, B, D–F). This suggests a positive regulatory role of SIX1 on these three proteins. Additionally, CD44 exhibited decreased mRNA levels upon Six1 knockdown in 66cl4 cells, while its expression increased upon SIX1 overexpression in MCF7 cells (Fig. 5B). However, no corresponding trend was observed at the protein level. Moreover, we observed decreased levels of ITGB1 and EPCAM proteins upon Six1 knockdown in 66cl4 cells, whereas their expression increased upon SIX1 overexpression in MCF7 cells (Fig. 5B). Furthermore, C-MYC and P-STAT3 displayed decreased expression levels upon Six1 knockdown in 66cl4 cells, while their levels increased upon SIX1 overexpression in MCF7 cells (Fig. 5B, H).

### SIX1 expression in breast cancer cells can influence the capacity of self‑renewal and proliferation in vitro

**Fig. 6:**
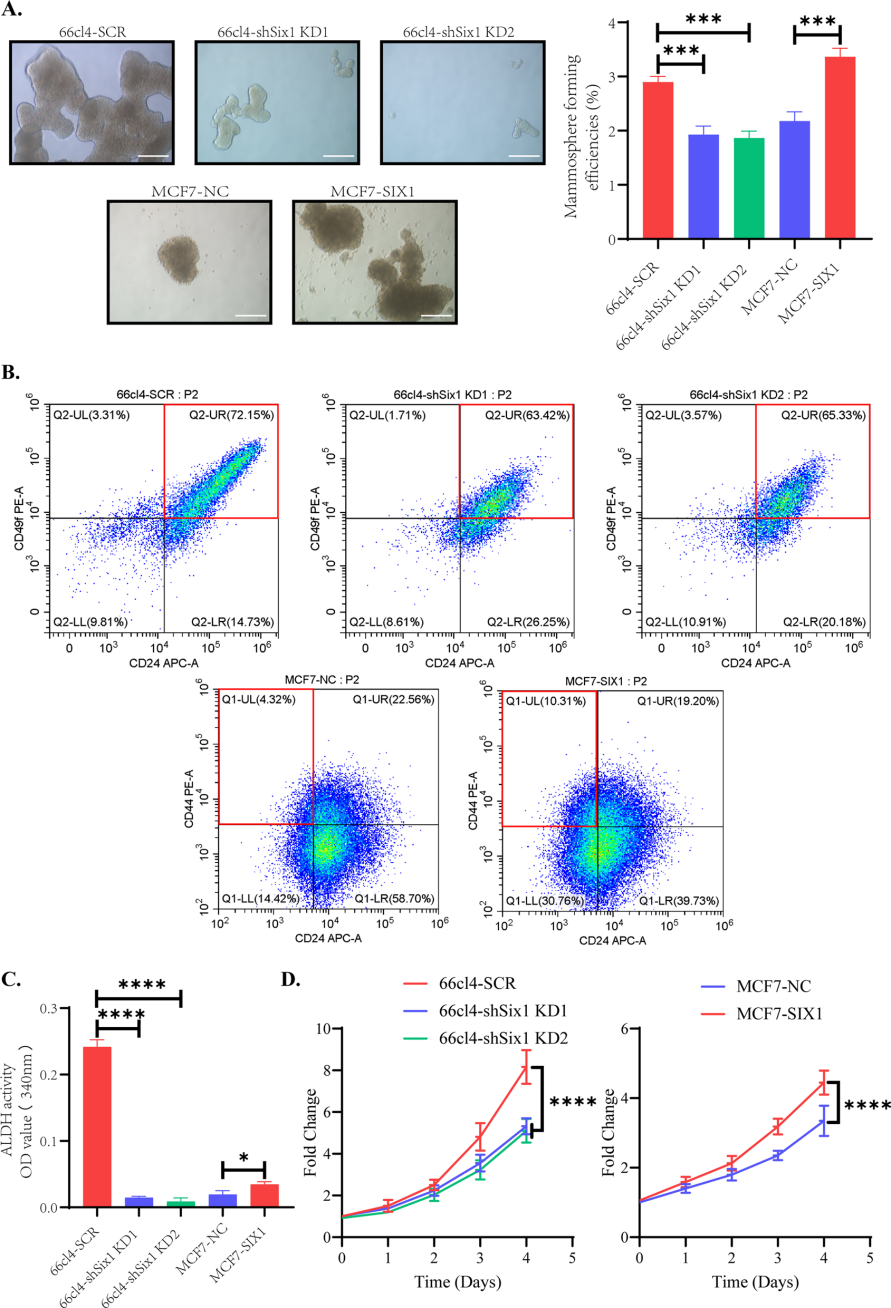
Changes in stem cell-related phenotypes of breast cancer cells following altered expression levels of SIX1. **A** Representative images of mammospheres and their corresponding mammosphere-formation efficiencies in 66cl4-SCR, 66cl4-shSix1 KD1, 66cl4-shSix1 KD2, MCF-7-NC, and MCF-7-SIX1 cells. The images were captured at a magnification of × 100, and the scale bar represents 200 µm. The data are presented as the mean ± standard deviation (SD) (n = 3). **B** Flow cytometry analysis of CD24 and CD49f expression in 66cl4-SCR, 66cl4-shSix1 KD1, and 66cl4-shSix1 KD2 cells, as well as CD24 and CD44 expression in MCF-7-NC and MCF-7-SIX1 cells. The red boxes indicate the stem cell population. **C** Bar graph showing the activity of ALDH enzyme in 66cl4-SCR, 66cl4-shSix1 KD1, 66cl4-shSix1 KD2, MCF-7-NC, and MCF-7-SIX1 cells. Higher OD values indicates stronger activity (n = 3). **D** Cell growth of 66cl4-SCR, 66cl4-shSix1 KD1, 66cl4-shSix1 KD2, MCF-7-NC, and MCF-7-SIX1 cells cultured for 0, 24, 48, 72, and 96 h (n = 4). Statistical significance is denoted as *P < 0.05, **P < 0.01, ***P < 0.001, ****P < 0.0001 respectively, compared to the control group

Based on our aforementioned experiments, we have observed that SIX1 effectively regulates genes and proteins associated with stem cells in breast cancer cell lines. Therefore, we aimed to investigate whether SIX1 can modulate breast cancer stem cells at a phenotypic level. Firstly, a mammosphere assay was conducted to assess the stemness of tumor cells. It was observed that the size of mammospheres and their forming efficiencies were positively correlated with the strength of stemness. Notably, efficient knockdown of Six1 resulted in reduced mammosphere sizes and diminished self-renewal capacity, as illustrated in Fig. 6A. Conversely, overexpression of SIX1 in MCF-7 cells significantly increased both the size of mammospheres and their forming efficiencies (Fig. 6A).

Moreover, recent studies suggest that CD24 + /CD49f + murine breast cancer cells and CD24-/CD44 + human breast cancer cells exhibit stem cell properties, also known as breast cancer stem cells or breast cancer stem-like cells [15, 29]. Therefore, in murine breast cancer cells, a higher proportion of CD24 + /CD49f + subpopulation indicates stronger stemness, while a lower proportion of CD24 + /CD49f + subpopulation suggests weaker stemness. Similarly, in human-derived breast cancer cells, a higher proportion of CD24-/CD44 + subpopulation indicates stronger stemness, while a lower proportion of CD24-/CD44 + subpopulation suggests weaker stemness. As shown in Fig. 6B, efficient knockdown of Six1 resulted in a reduction of the CD24 + /CD49f + subpopulation, whereas overexpression of SIX1 in MCF-7 cells led to an increase in the CD24-/CD44 + subpopulation.

Furthermore, ALDH is an essential enzyme involved in important cellular mechanisms such as aldehyde detoxification and retinoic acid synthesis, and its activity is linked to drug resistance—a characteristic of cancer stem cells [30, 31]. Therefore, we sought to demonstrate the ability of SIX1 to influence breast cancer stem cells by assessing its effect on ALDH activity. In Fig. 6C, efficient knockdown of Six1 substantially reduced the activity of ALDH enzyme, while overexpression of SIX1 in MCF-7 increased ALDH activity (Fig. 6C).

Finally, we examined the proliferative rate of cancer cells upon alteration of SIX1 expression. Notably, efficient knockdown of Six1 reduced the rate of cancer cell proliferation, which became more pronounced over time, as shown in Fig. 6D. Conversely, overexpression of SIX1 in MCF-7 cells led to a greater increase in cancer cell proliferation over time (Fig. 6D). Taken together, these findings demonstrate that SIX1 positively modulates breast cancer stem cells at a phenotypic level, implicating its potential as a therapeutic target in breast cancer treatment.

### SIX1 expression in breast cancer cells can influence the capacity of self‑renewal and proliferation in vivo

**Fig. 7:**
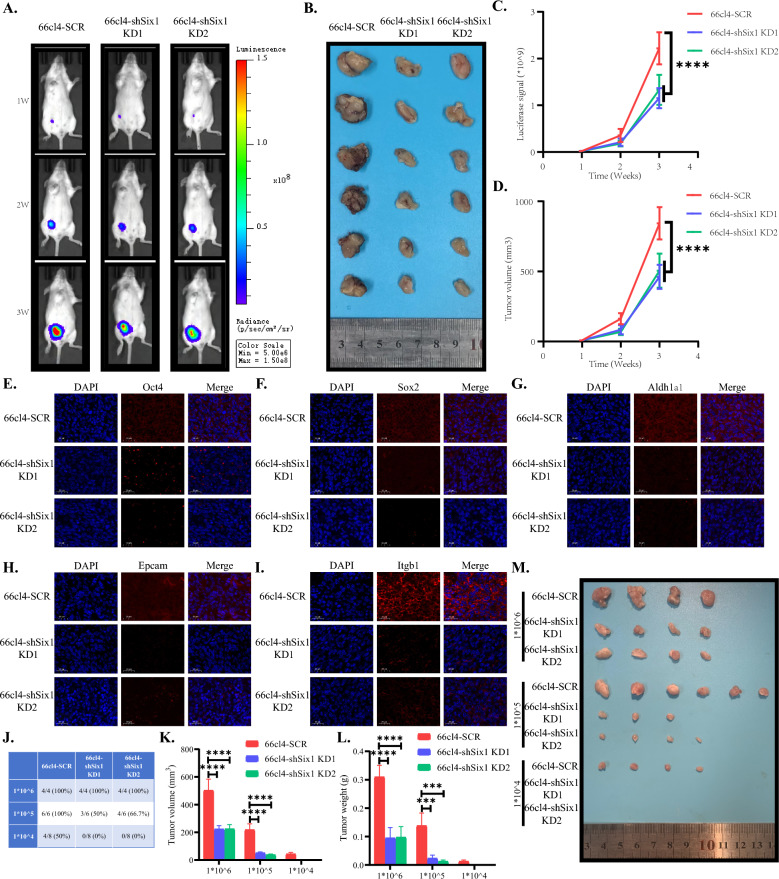
In vitro alterations in tumor growth rate, stem cell marker expression and tumorigenic capacity in response to modulation of SIX1 expression levels. **A** Luminescence imaging of 66cl4-SCR-luc, 66cl4-shSix1 KD1-luc, and 66cl4-shSix1 KD2-luc tumor-bearing mice on week 1, 2, and 3 (n = 6); **B** Photograph of dissected tumors (n = 6); **C** Quantitation of luminescent signal of 66cl4-SCR-luc, 66cl4-shSix1 KD1-luc, and 66cl4-shSix1 KD2-luc tumor-bearing mice (n = 6); **D** Tumor growth curves of 66cl4-SCR-luc, 66cl4-shSix1 KD1-luc, and 66cl4-shSix1 KD2-luc tumor-bearing mice (n = 6). **E**–**I** Representative immunofluorescent images of Oct4, Sox2, Aldh1a1, Epcam, and Itgb1 in tumor tissues generated by 66cl4-SCR-luc, 66cl4-shSix1 KD1-luc, and 66cl4-shSix1 KD2-luc cells. **J** Tumor formation rates of BALB/C mice at 6–8 weeks after injecting different amounts of 66cl4-SCR-luc, 66cl4-shSix1 KD1-luc, and 66cl4-shSix1 KD2-luc cells into the fat pad of BALB/C mice for 10 days are shown in a table (n = 4–8). **K** Tumor volumes at the end of the experiment are presented (n = 4–8). **L** Tumor weights at the end of the experiment are provided (n = 4–8). **M** Tumors harvested at the end of the experiment are depicted (n = 4–8)

We have presented evidence demonstrating the impact of SIX1 on the self-renewal and proliferation abilities of breast cancer cells in vitro. Our subsequent investigations aim to further characterize the role of SIX1 under in vivo conditions. To this end, we employed BALB/c mice (6–8 weeks old) and randomly divided them into three distinct groups. The three groups of mice were utilized to establish tumor models of 66cl4-SCR-luc, 66cl4-shSix1 KD1-luc, and 66cl4-shSix1 KD2-luc, respectively. We employed two methods to determine tumor volume in the mice. The first method involved measuring bioluminescence intensity by IVIS imaging. Prior to imaging, 300 µL of D-luciferin potassium salt buffer solution with a 15 mg/mL concentration was intraperitoneally injected into each mouse and incubated for 9 min to maximize the bioluminescence signal intensity (Fig. 7A). The second method involved calculating tumor volume at 7 day intervals up to the end of the experiment using the following formula: width2 × length × 0.5. After three weeks, all mice were euthanized, and tumors were removed (Fig. 7B). Figure 7C, D showed the luciferase signal curve and tumor volume of tumor, respectively (Fig. 7D). We observed that the tumors of 66cl4-SCR-luc bearing mice were larger and grew faster compared to those of 66cl4-shSix1 KD1-luc and 66cl4-shSix1 KD2-luc bearing mice. This difference became more pronounced over time, indicating a decrease in malignancy after the knocking down of Six1. We performed immunofluorescence on tumor samples embedded in paraffin to investigate the impact of SIX1 on the expression of stem cell-related markers in tumor tissues. We observed varying degrees of decrease in the fluorescence intensity of Oct4, Sox2, Aldh1a1, Epcam, and Itgb1 in tumor tissues formed by 66cl4-shSix1 KD1-luc and 66cl4-shSix1 KD2-luc compared to those formed by 66cl4-SCR-luc. This suggests that the decreased expression of Six1 correlates with a decrease in the expression of Oct4, Sox2, Aldh1a1, Epcam, and Itgb1 at both the tissue and cellular levels (Fig. 7E–I). To further confirm the role of Six1 in enhancing the stemness of breast cancer cells, we injected different amounts of 66cl4-SCR-luc, 66cl4-shSix1 KD1-luc, and 66cl4-shSix1 KD2-luc cells to assess their tumorigenic ability. Cells with a higher capacity for tumor formation exhibit stronger stemness even at lower cell numbers. When 1*10^6^ cells were injected, all groups formed tumors. At 1*10^5^ cells, the tumor formation efficiency was 100% in the 66cl4-SCR-luc group, 50% in the 66cl4-shSix1 KD1-luc group, and 66.7% in the 66cl4-shSix1 KD2-luc group. When the number of injected cells was further reduced to 1*10^4^, the tumor formation efficiency was 50% in the 66cl4-SCR-luc group, while it was 0% in both the 66cl4-shSix1 KD1-luc and 66cl4-shSix1 KD2-luc groups (Fig. 7J). Additionally, regardless of the number of cells injected, the tumor volume and size were larger in the 66cl4-SCR-luc group compared to the other two groups (Fig. 7K, L). All tumors are depicted in Fig. 7M. These findings further validate that the expression of Six1 can increase the stemness of breast cancer cells in vivo.

## Disscussion

In conclusion, we have employed online databases to elucidate the modifications in SIX1 mRNA expression and its correlation with prognosis, ploidy, immune infiltration, gene alteration landscape, mRNA and protein landscape, altered signal pathways of SIX1, and particularly, the biomarkers of cancer stem cells during the development of breast cancer. Additionally, we have conducted both in vitro and in vivo experiments to demonstrate the regulatory role of SIX1 in breast cancer stem cells.

Our findings indicate that SIX1 mRNA expression is upregulated in patients with breast cancer and is associated with different subtypes of the disease. Compared to the normal group, all four major breast cancer subtypes exhibited increased expression of SIX1, with the luminal B subtype showing the most significant increase. Previous studies by Heide L Ford et al. have shown that elevated SIX1 expression is correlated with poor prognosis in luminal breast cancers [17]. Consistent with these findings, our study revealed that within the luminal subtype, patients with high SIX1 expression had a worse prognosis compared to those with low expression. Surprisingly, we also observed a similar trend in two other subtypes of breast cancer, namely HER2 positive and triple-negative breast cancer (TNBC), where patients with high SIX1 expression had a poorer prognosis compared to those with low expression. This novel finding has not been reported in previous studies. As an established hallmark of cancer, approximately 75% of solid tumors exhibit aneuploidy and chromosomal instability, resulting in a complex and heterogeneous karyotypic landscape [32, 33]. Assessing tumor ploidy provides valuable insights into cancer genome evolution and tumor heterogeneity [32, 33]. In our study, we discovered a positive association between SIX1 expression and tumor ploidy in breast cancer. This suggests that SIX1 is closely involved in polyploidy and chromosomal instability in breast cancer, further enhancing our understanding of its role in disease development. Moreover, research has demonstrated that SIX1 plays a unique role in the breast cancer microenvironment. Micalizzi et al. have shown that SIX1 promotes the recruitment of tumor-associated macrophages (TAMs) and cancer-associated fibroblasts (CAFs) [34]. Similarly, our results reveal that as SIX1 expression increases, there is a decrease in overall immune cell infiltration and an increase in M2 macrophages and Treg cells, potentially leading to the suppression of other immune cells and favoring tumor cell growth. Additionally, our study found that amplification of mRNA expression or mutations account for most alterations in SIX1 during breast cancer tumorigenesis.

As a transcription factor and proto-oncogene, SIX1 can activate and inhibit transcription. SIX1 lacks an activation domain and requires cofactors to exert its effects. For transcriptional activation, SIX1 interacts with EYA family proteins and mediates their nuclear translocation to exert its effects [35, 36]. For transcriptional repression, SIX1 acts synergistically with DACH family proteins and represses downstream targets [35, 36]. SIX1 has been reported to play a role in the development of muscles, kidneys, craniofacial structures, and sense organs, which aligns with our enrichment results of GO [10]. In the early stages of development, SIX1 is vital for the expansion of progenitor cell populations and intercellular communication, mediating tissue and organ development [10, 12]. Furthermore, our KEGG enrichment analysis suggests that SIX1 may be involved in multiple critical signaling pathways such as WNT, estrogen, MAPK, PI3K-Akt, and IL17 pathways, providing new insights into the underlying mechanisms of SIX1 function.

Recent research has focused on the relationship and mechanism of SIX1 in regulating breast cancer stem cells. SIX1 has been reported to increase CSC numbers in vitro and in vivo: SIX1 increased phenotypic and functional CSCs in breast [17], colorectal [18] and esophageal cancer [19] and phenotypic CSCs in pancreatic cancers [20]. Six1 has been reported to increase tumor initiating cells by WNT, MAPK and transforming growth factor-beta (TGF-β) signaling pathways [17, 37]. In this study, our investigations, encompassing both in vitro and in vivo experiments, have substantiated the regulatory capacity of SIX1 on breast cancer stem cells. A noteworthy aspect of our research lies in the utilization of the previously unexplored TNBC cell line 66cl4 as a novel model for examining the mechanisms underlying SIX1's actions in breast cancer. Moreover, we bolstered our findings by employing an additional cell model derived from the luminal cell line MCF-7, thereby enhancing the reliability of our results. Precisely, we noted a positive correlation between changes in SIX1 expression levels and increased mammosphere sizes, mammosphere-forming efficiencies, the proportion of stem cells (CD24 + /CD49f + , CD24-/CD44 +), ALDH activity, as well as in vitro proliferation.

In vivo experiments were conducted to investigate the role of Six1 in breast cancer. Knocking down Six1 was found to reduce the growth rate of tumors, with the effect becoming more pronounced over time. Additionally, reducing the number of tumor cells injected continuously demonstrated a decrease in the tumorigenic ability of breast cancer cells after Six1 knockdown. These findings suggest that Six1 expression promotes the formation and growth of breast cancer masses, and that the formation and growth of breast cancer is indicative of its stemness [38, 39].

Previous studies have shown that OCT4 [40], SOX2 [41], ALDH1A1 [42], EPCAM [43], and ITGB1 [44] positively regulate stemness in cancer. Both in vitro and in vivo experiments confirmed that Six1 positively regulates OCT4, SOX2, ALDH1A1, EPCAM, and ITGB1. Similarly, changes in p-STAT3 were observed, indicating that the STAT3 signaling pathway may also be involved in the regulation of breast cancer stem cells by Six1. Further research in this direction will be pursued in future studies. The specific regulatory mechanism remains unclear. It is possible that Six1 promotes translation by enhancing the transcription of OCT4, SOX2, ALDH1A1, ultimately leading to enhanced stemness. Moreover, Six1 may promote EPCAM and ITGB1 function enhancement, which in turn enhances stemness through protein–protein interactions. Further research is required to fully elucidate these mechanisms. In our previous work on thyroid cancer [45], we found that Six1 activates the STAT3 signaling pathway via EYA1, resulting in increased expression of p-STAT3 and C-MYC. In this study on breast cancer, we also observed increased expression of p-STAT3 and C-MYC, suggesting that Six1's regulation of breast cancer stem cells may also involve the STAT3 signaling pathway. Investigating how Six1 regulates OCT4, SOX2, ALDH1A1, EPCAM, and ITGB1, as well as its potential activation of the STAT3 signaling pathway, will be the focus of our future experiments.

During embryonic development, the process of EMT plays a defining role in proper body patterning and morphogenesis. In cancer, SIX1 overexpression induces EMT in a wide range of cancer cell types, including those derived from breast tissue [46]. EMT not only facilitates cancer metastasis, but also drives an increase in CSC populations, as determined by both surface marker expression and enrichment for tumor initiating cells [47]. Furthermore, SIX1 overexpression enhances self-renewal capacity and promotes EMT in breast cancer cells, contributing to a stem-like phenotype. Therefore, the interplay between SIX1, EMT, and CSCs must be considered in future investigations, particularly in the context of mechanisms governing the effects of SIX1 on breast cancer pathobiology, with the goal of identifying new therapeutic targets for breast cancer patients [46, 48].

## Conclusion

In summary, the distinct expression pattern, transcriptional profile, involvement in ca, as well as interaction with cancer stem cells, present a fresh perspective for targeted molecular therapy of SIX1 in breast cancer patients and offer leads and insights towards a deeper understanding of the molecular mechanisms driving breast cancer tumorigenesis. Further investigation is required to explore alternate modes of regulating breast cancer stem cells by SIX1.

### Supplementary Information

**Additional file 1: Table S1.** Detailed information of differential expressed genes. Detailed information of differential expressed genes between the high SIX1 expression group and the low SIX1 expression group. The experimental group comprising patients in the top 25% and the control group comprising patients in the bottom 25%.

## Data Availability

Data are available from the corresponding author upon reasonable request.

## Abbreviations

SIX1: Sine oculis homeobox homolog 1
CSCs: Cancer stem cells
BCSCs: Breast cancer stem cells
OS: Overall survival
TNBC: Triple negative breast cancer
GO: Gene Ontology
KEGG: Kyoto Encyclopedia of Genes and Genomes
DMEM: Dulbecco’s modified Eagle’s medium
RT-qPCR: Quantitative real-time PCR
ALDH: Aldehyde dehydrogenase
OD: Optical density
CCK-8: Cell Counting Kit 8
IDC: Invasive ductal carcinoma
ILC: Invasive lobular carcinoma
EMT: Epithelial-mesenchymal transition
HD: Homeodomain
SD: SIX domain
TAMs: Tumor-associated macrophages
CAFs: Cancer-associated fibroblasts
SD: Standard deviation
